# First Complete *Providencia rettgeri* Genome Sequence, the NDM-1-Producing Clinical Strain RB151

**DOI:** 10.1128/genomeA.01472-16

**Published:** 2017-01-19

**Authors:** R. Alejandro Marquez-Ortiz, Leanne Haggerty, Eby M. Sim, Carolina Duarte, Betsy E. Castro-Cardozo, Mauricio Beltran, Sandra Saavedra, Natasha Vanegas, Javier Escobar-Perez, Nicola K. Petty

**Affiliations:** a Bacterial Molecular Genetics Laboratory, Universidad El Bosque, Bogotá, Colombia; b The ithree Institute, University of Technology Sydney, Sydney, Australia; c Grupo de Microbiología, Instituto Nacional de Salud, Bogotá, Colombia

## Abstract

*Providencia rettgeri* is an opportunistic bacterial pathogen of clinical significance due to its association with urinary tract infections and multidrug resistance. Here, we report the first complete genome sequence of *P. rettgeri*. The genome of strain RB151 consists of a 4.8-Mbp chromosome and a 108-kbp *bla*_NDM-1_-positive plasmid.

## GENOME ANNOUNCEMENT

*Providencia rettgeri* is an opportunistic human pathogen mainly associated with urinary tract infections (1, 2). A Gram-negative member of the *Enterobacteriaceae*, *P. rettgeri* is also known to cause diarrhea, meningitis, eye infections, and bacteremia in both hospital and community settings (1–5). *P. rettgeri* is intrinsically resistant to several antibiotics (5, 6), but notably, many recent independent isolates have been found to be carbapenemase producers carrying the New Delhi metallo-β-lactamase (NDM) gene *bla*_NDM-1_ (7–9). To date, there are only a few draft genomes of *P. rettgeri* available in the public databases. Here, we report the first complete genome sequence of *P. rettgeri*, that of a multidrug-resistant clinical isolate carrying the *bla*_NDM-1_ gene.

*P. rettgeri* RB151 was isolated in 2013 from a urine sample from a 58-year-old female patient, diagnosed with a urinary tract infection in the emergency department of a tertiary hospital in Bucaramanga, Colombia (7). Total genomic DNA was extracted using the UltraClean microbial DNA isolation kit (Mo Bio Laboratories, Inc.). A 20-kb BluePippin (Sage Science) size-selected SMRTbell library was constructed and sequenced using one single-molecule real-time (SMRT) cell with P6-C4 chemistry on the PacBio RSII platform (Pacific Biosciences, CA). The resulting 167,518 reads, which had an *N*_50_ read length of 10,167 bp, were *de novo* assembled using the RS_HGAP_Assembly.3 protocol implemented in SMRT Analysis version 2.3 (10), into two contigs with a total genome length of 4.9 Mbp. The filtered subreads were then mapped to the assembly using BWA-MEM (11), revealing an average coverage of 176×. The assembly was manually checked using Tablet (12), and low-coverage misassembled terminal repeat sequences, a known artifact of HGAP assembly (10, 13), were manually trimmed and removed from each contig. The final sequences were manually reordered so that the linear representation of each circular contig started at *dnaA* (chromosome) and *repA* (plasmid). The final assembly was verified using Circlator version 1.4.0 (13) and Artemis Comparison Tool version 13 (14). The genome was annotated using Prokka version 1.11 (15), and the antibiotic resistance genes were identified using ARIBA (https://github.com/sanger-pathogens/ariba).

The complete genome of *P. rettgeri* RB151 has an average G+C content of 41.7% and consists of a 4,780,676-bp chromosome and a 108,417-bp NDM-1-encoding plasmid (pRB151-NDM). The automated genome annotation predicted 4,497 coding sequences (CDSs), 22 rRNAs, 77 tRNAs, and one transfer-messenger RNA (tmRNA). The antimicrobial resistome of the RB151 chromosome evaluated using ARIBA included resistance genes to aminoglycosides [*aac(3)-Iia*, *armA,* and *aacA4*], β-lactams (*bla*_TEM-1B_ and *bla*_OXA-2_), fluoroquinolones [*aac(6′)Ib-cr*], sulfonamides (*sul1* and *sul2*), and trimethoprim (*dfrA31*). The plasmid pRB151-NDM only contained the *bla*_NDM-1_ gene, which confers resistance to β-lactams.

As the first complete genome of *P. rettgeri*, this genome sequence will be a useful reference genome and could be utilized to contribute further insights into this species.

### Accession number(s).

The complete genome of *Providencia rettgeri* RB151 has been deposited in DDBJ/EMBL/GenBank under the GenBank accession numbers CP017671 (chromosome) and CP017672 (plasmid pRB151-NDM). The version described in this paper is the first version.
